# Synthesis of One-Dimensional Mesoporous Ag Nanoparticles-Modified TiO_2_ Nanofibers by Electrospinning for Lithium Ion Batteries

**DOI:** 10.3390/ma12162630

**Published:** 2019-08-18

**Authors:** Yuyao Zhang, Jun Li, Wenyao Li, Danning Kang

**Affiliations:** School of Materials Engineering, Shanghai University of Engineering Science, Shanghai 201620, China

**Keywords:** electrospinning, titanium oxide, sliver, mesoporous nanofibers, lithium ion battery

## Abstract

TiO_2_ is regarded as a prospective electrode material owing to its excellent electrochemical properties such as the excellent cycling stability and the high safety. However, its low capacity and low electronic conductivity greatly restrict the further improvement in electrochemical performance. A new strategy was put forward to solve the above defects involved in TiO_2_ in which the low capacity was enhanced by nanomerization and porosity of TiO_2_, and the low electronic conductivity was improved by introducing Ag with a high conductivity. One-dimensional mesoporous Ag nanoparticles-embedded TiO_2_ nanofibers (Ag@TiO_2_ nanofibers) were successfully synthesized via a one-step electrospinning process combined with subsequent annealing treatment in this study. The microstructure and morphology of mesoporous TiO_2_@Ag nanofibers were confirmed by X-ray diffraction, scanning electron microscopy, transmission electron microscopy, and nitrogen adsorption–desorption. TiO_2_ nanofibers mainly consisted of a large amount of anatase TiO_2_, accompanied with traces of rutile TiO_2_. Ag nanoparticles were uniformly distributed throughout TiO_2_ nanofibers and promoted the transformation of TiO_2_ from the anatase to the rutile. The corresponding electrochemical performances are measured by galvanostatic charge-discharge, cycle stability, rate performance, cycle voltammetry, and electrochemical impedance spectroscopy measurements in this research, with pristine TiO_2_ nanofibers as the reference. The results indicated that the introduction of Ag nanoparticles into TiO_2_ nanofibers significantly improved the diffusion coefficient of Li ions (5.42 × 10^−9^ cm^2^⋅s^−1^ for pristine TiO_2_, 1.96 × 10^−8^ cm^2^⋅s^−1^ for Ag@TiO_2_), and the electronic conductivity of TiO_2_ (1.69 × 10^−5^ S⋅cm^−1^ for pristine TiO_2_, and 1.99 × 10^−5^ S⋅cm^−1^ for Ag@TiO_2_), based on which the comprehensive electrochemical performance were greatly enhanced. The coulombic efficiency of the Ag@TiO_2_ nanofibers electrode at the first three cycles was about 56%, 93%, and 96%, which was higher than that without Ag (48%, 66%, and 79%). The Ag@TiO_2_ nanofibers electrode exhibited a higher specific discharge capacity of about 128.23 mAh⋅g^−1^ when compared with that without Ag (72.76 mAh·g^−1^) after 100 cycles at 100 mA·g^−1^. With the current density sharply increased from 40 mA·g^−1^ to 1000 mA·g^−1^, the higher average discharge capacity of 56.35 mAh·g^−1^ was remained in the electrode with Ag, when compared with the electrode without Ag (average discharge capacity of about 12.14 mAh·g^−1^). When the current density was returned to 40 mA·g^−1^, 80.36% of the initial value was returned (about 162.25 mAh·g^−1^) in the electrode with Ag, which was evidently superior to that without Ag (about 86.50 mAh·g^−1^, only 55.42% of the initial value). One-dimensional mesoporous Ag@TiO_2_ nanofibers can be regarded as a potential and promising candidate as anode materials for lithium ion batteries.

## 1. Introduction

Li-ion batteries as a kind of rechargeable device have been widely used owing to their large specific capacity, long cycle life, low self-discharge rate, and so on [1]. The anode materials play an essential role in their electrochemical performance. The commercial graphitic carbon has been applied as the anode material due to its low cost, high abundance, and outstanding kinetics. However, some shortcomings involved in carbon severely restrict the further improvement in electrochemical performance of Li-ion batteries. Carbon will suffer from the easy formation of solid electrolyte interface (SEI), which results in poor rate performance [2]. Accompanied with that, the capacity is greatly reduced with prolonging the service life. Moreover, the hazardous Li dendrites are also subject to being formed in graphite anode with a very low Li-intercalation potential of about 0 V (vs Li^+^/Li) during the overcharge, which greatly increases the potential risks of explosion and fire. Therefore, it is an urgent problem to explore a new material with high capacity and excellent cycle performance as a substitute for commercial graphite electrode.

Transition metal oxides (such as TiO_2_ [3], MnO_2_ [4], Co_3_O_4_ [5], V_2_O_5_ [6], SnO_2_ [7], and NiO [8]) have been widely considered as attractive electrode substitutes. Among them, titanium oxide (TiO_2_) was a potential candidate as anode materials for lithium ion batteries [9], which has also been used in many other fields, like solar cell [10], biosensors [11], photocatalysis [12], etc. There are four common polymorphs of TiO_2_: Rutile, anatase, brookite [13], and TiO_2_-B [14], among which a large number of investigations into anatase TiO_2_ as a prospective electrode material have been carried out due to its superb electrochemical properties such as the excellent cycling stability and the high safety. The former is attributed to the small volume expansion (3–4%) of anatase TiO_2_ during charging and discharging [15]. The latter is closely associated with the high voltage of discharge platform (~1.78 V vs Li^+^/Li) of anatase TiO_2_ [16]. This voltage is significantly higher than that of graphitic anodes (~0 V vs Li^+^/Li), which effectively avoids the formation of hazardous Li dendrites. However, a low capacity (335 mAh·g^−1^) [17] and a low electronic conductivity of TiO_2_ (10^−7^–10^−9^ S·cm^−1^) greatly reduces the charge transportation [18], which restricts the further improvement in capacity and rate capability. Moreover, the capacity retention is also greatly limited due to the large irreversible capacity loss during the first cycling, which further deteriorates the electrochemical performance [19]. 

The above-mentioned defects involved in TiO_2_ can be overcome to a certain extent by a unique structural design, namely fabricating porous nanosized TiO_2_ and introducing the other elements with a high conductivity, and so on. The former strategy can significantly enlarge the electrode/electrolyte interfacial area, and shorten lithium diffusion path, which will further improve the specific capacity and rate capability. Armstrong et al. [20] prepared TiO_2_ nanowires by hydrothermal reaction followed by annealing and compared their electrochemical performance with that of bulk TiO_2_. The result indicated that TiO_2_ nanowires exhibited a higher specific capacity of 305 mAh⋅g^−1^ than that of bulk TiO_2_ (240 mAh·g^−1^). Bao et al. [21] successfully synthesized porous anatase TiO_2_ nanorods by a simple approach based on a reaction in composite-hydroxide eutectic system without using an organic dispersant or capping agent using a binary eutectic mixture system. A network pore structure was formed among a large number of small interconnected nanoparticles involved in nanorods. TiO_2_ nanorods with the unique porous structure were endowed with more excellent electrochemical performance when compared with TiO_2_ nanopowders with poor pore structure. Initial discharge capacity (266.4 mAh·g^−1^ at 60 mA·g^−1^) of porous TiO_2_ nanorods was higher than that of TiO_2_ nanopowders (150 mAh·g^−1^). Moreover, capacity retention (170 mAh·g^−1^) was obtained in porous TiO_2_ nanorods, which was more than twice that of TiO_2_ nanopowders after 30 cycles. Therefore, the nanomerization and porosity of TiO_2_ will contribute to the improvement in electrochemical performance of TiO_2_. In addition to those, the latter also contributes to the improvement in rate capability due to the formation of a conductive percolation network. Opra et al. [22] adopted sol-gel template method to utilize Zr^4+^/F^−^ doped TiO_2_ nanotube as anode material for lithium ion battery, the electrochemical performance of Ti_0.97_Zr_0.03_O_1.98_F_0.02_ was enhanced by the increasing electronic conductivity of F^−^ incorporation, which has been calculated in detail. It exhibited reversible capacity (~163 mAh·g^-1^ at 1 C) and rate capacity (~138 mAh·g^−1^ at 10 C). Fehse et al. [23] fabricate the Nb-doped TiO_2_ nanofibers by electrospinning used as anode material. Although the cycling performances did not make a large difference, when compared the rate capability with non-doped TiO_2_ nanofibers, it showed better performance (~23 mAh·g^−1^ at 5 C, about twice than non-doped TiO_2_) caused by the enhancement of electronic conductivity. Electrospinning is regarded as a versatile method for fabricating continuous 1D nanofibers with a large specific surface area [24]. Many investigations into the preparation of 1D TiO_2_ nanofibers decorated with the other materials have been carried out. Yang et al. [25] synthesized carbon@TiO_2_ composite nanofibers through electrospinning followed by a subsequent annealing treatment. These hybrid nanofibers maximized the advantage of each material, which provided sufficient electrode-electrolyte contacts and short lithium ion diffusion pathways during discharge/charge cycling, and thus made a great contribution to lithium storage capacity. The electrode displayed a high initial reversible capacity of 217.1 mAh⋅g^−1^ with high coulombic efficiency of nearly 100% at the current density of 30 mA⋅g^−1^ and still maintained a reversible capacity of approximately 206 mAh⋅g^−1^ with 100% coulombic efficiency after 100 cycles. Han et al. [26] prepared nitridated TiO_2_ hollow nanofibers using a simple electrospinning method and subsequent nitridation treatment. The electrode exhibited the first discharge capacity of about 180 mAh⋅g^−1^ at 0.2 C, and the initial coulombic efficiency of 86.8%. Its rate capacity was 50 mAh⋅g^−1^ at 5 C, which was twice higher than that of pristine TiO_2_ nanofibers electrode. This was mainly attributed to shorter lithium ion diffusion length and high electronic conductivity along the surface of nitridated hollow nanofibers. Li et al. [27] synthesized boron-doping anatase TiO_2_ nanofibers via the combination of electrospinning and annealing. The incorporation of B element promoted the crystallization of the building subunits of microporous TiO_2_, which was beneficial to the improvement in electrochemical performance at higher current rates and longer cycles. The electrode demonstrated a capacity of 147 mAh⋅g^−1^ at a current density 4 A⋅g^−1^ and an excellent long-term cycling stability with a capacity retention of 167.6 mAh⋅g^−1^ at 2 A⋅g^−1^ over 5000 cycles. Tran et al. [28] incorporated nanostructured SnO_2_ with an impressive theoretical capacity of 781 mAh⋅g^−1^ into TiO_2_ nanofibers matrix using a facile and low-cost electrospinning technique combined with a sol-gel method, followed by annealing treatment. The SnO_2_@TiO_2_ composite nanofibers electrode possessed a much higher initial specific capacity of about 560 mAh⋅g^−1^ at 100 mA⋅g^−1^ and a capacity retention of about 5% at 100 mA⋅g^−1^ after 50 cycles. Wu et al. [29] fabricated nanosized Si/C/TiO_2_ composite nanofibers through the electrospinning and annealing method, which was a promising candidate for lithium ion battery anode because of its high theoretical capacity (1200 mAh⋅g^−1^) and stable cycling performance (600 cycles). A much higher gravimetric specific capacity as high as 720 mAh⋅g^−1^ at 48 mA⋅g^−1^ can be acquired in the composite electrode (only 83 mAh⋅g^−1^ for pure TiO_2_ nanofibers electrode), accompanied with more than 94% capacity after 55 cycles. Zhou et al. [30] prepared MoS_2_ nanograins doped TiO_2_ nanofibers via the electrospinning and annealing. The electrode exhibited the initial discharge and charge capacities of 721.3 and 495.1 mAh⋅g^−1^. A stable specific capacity of 479.78 mAh⋅g^−1^ was maintained at 100 mA⋅g^−1^ after 100 cycles and a high rate capability was also obtained with increasing the current density (412, 303, and 216 mAh⋅g^−1^ at 200, 500, and 800 mA⋅g^−1^). Lee et al. [31] reported a methodology to control the crystal structure of TiO_2_ nanofibers by adding aluminum isopropoxide into a common sol-gel precursor solution, followed by electrospinning and annealing. The electrode prepared with anatase TiO_2_ nanofibers exhibited an initial coulombic efficiency of 83.9%, a stable specific capacity of 150 mAh⋅g^−1^ at 40 mA⋅g^−1^ after 100 cycles, and a high rate capability of 48.5% at 2000 mA⋅g^−1^. Nam et al. [32] prepared Au nanoparticle-embedded TiO_2_ nanofibers via a one-step electrospinning process and followed a heat treatment. Au nanoparticles as conductive agents were embedded into the electrochemically active TiO_2_ matrix. In comparison to pristine TiO_2_ electrode, the specific capacity of the electrode with Au nanoparticle was about 150 mAh⋅g^−1^ at 66 mA⋅g^−1^ after 50 cycles, increased by at least 20%. The rate performance for Au@TiO_2_ electrode has enhanced 30% at 0.1 C (170 mAh⋅g^−1^), at least 2-fold at 2 C (70 mAh⋅g^−1^), and 24-fold at 5 C (45 mAh⋅g^−1^) when compared to sample without Au nanoparticles.

It has been proved that the incorporation of metal nanoparticles in TiO_2_ nanofibers can endow the electrode with higher electrical conductivity, which could enhance its comprehensive electrochemical performance. Ag with a high conductivity (~10^8^ S⋅cm^−1^) is regarded as a potential embedded-candidate for TiO_2_ [32]. Unfortunately, there are only few investigations into the preparation of Ag-modified TiO_2_ nanofibers as the anode material by electrospinning. Lin et al. [33] successfully synthesized a series of Ag-modified TiO_2_ nanowires (Ag-NWs@TiO_2_) film electrodes with hierarchical 3D nano-network structure via a facile hydrothermal process followed by the traditional silver mirror reaction. The initial discharge capacity and the first coulombic efficiency of NWs@TiO_2_ electrodes were 324.5 mAh·g^−1^ and 66.3% at 200 mA⋅g^−1^, while the values of Ag-NWs@TiO_2_ electrodes were increased to 351.2 mAh⋅g^−1^ and 63.9%, respectively. Meng et al. [34] prepared three-dimensional (3D) ordered Ag nanoparticles-modified TiO_2_ nanotube arrays via facile electrodeposition. After 50 charge/discharge cycles, the capacity retention of composite Ag/TiO_2_ nanotubes electrode was about 94% of the initial discharge capacity, higher than 87.3% of bare TiO_2_ nanotubes electrode. As for rate capability, the discharge capacities of Ag/TiO_2_ nanotubes electrode were 110, 105, 100, 90 mAh⋅g^−1^ at the rate of 0.3 C, 0.6 C, 1.2 C, and 2.4 C, which were twice than those of bare TiO_2_ nanotubes electrode. The present limited investigations into the Ag-modified nanostructured TiO_2_ have proved the positive role resulting from the introduction of Ag. However, some shortcomings involved in present researches need to be improved. Ag nanoparticles are deposited on the surfaces of nanostructured TiO_2_, which will accelerate the transportation of charges among nanostructured TiO_2_. However, no Ag nanoparticles are embedded into the inner of nanastructured TiO_2_, which has no positive role in promoting the transportation of charges in the inner of independent nanostructured TiO_2_. Therefore, Ag nanoparticles dispersedly distributed within nanostructured TiO_2_ will be beneficial to the further improvement in electrochemical performance, accompanied with those adhering to the surfaces of nanostructured TiO_2_. 

In this research, the electrospinning technique followed by the annealing was applied to synthesize mesoporous Ag nanoparticles-embedded TiO_2_ (Ag@TiO_2_) nanofibers as LIB anodes. AgNO_3_ was used as Ag dopant source, and diisopropyl azodiformate (DIPA) was added into the precursor (tetra-n-butyl titanate and polyvinylpyrrolidone) to create a porous structure. X-ray diffraction (XRD), scanning electron microscopy (SEM), transmission electron microscopy (TEM), and X-ray photoelectron spectroscopy (XPS) were used to characterize the microstructure of TiO_2_ nanofibers with and without Ag nanoparticles. The porous properties of the nanofibers before and after modification were confirmed using nitrogen adsorption-desorption. The corresponding electrochemical performance was also compared with pristine TiO_2_ by galvanostatic charge-discharge and cycle voltammetry. All these methods verified that the introduction of Ag greatly improved the electrochemical performances of TiO_2_ nanofibers electrode. 

## 2. Experimental

### 2.1. Synthesis of Mesoporous TiO_2_ and Ag@TiO_2_ Nanofibers

**Figure 1:**
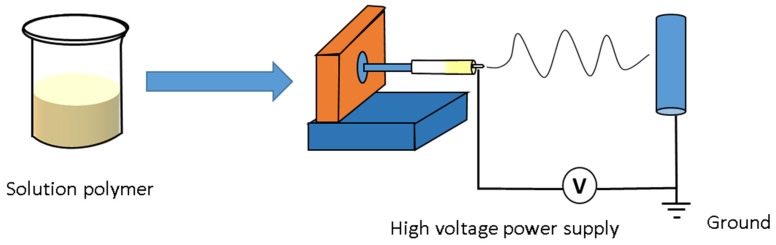
Schematic illustration of the electrospinning process.

Tetra-n-butyl titanate (TBOT, 99%, Sinopharm Chemical Reagent Co., Ltd, Shanghai, China), polyvinylpyrrolidone (PVP, M_W_ ≈ 1,300,000 g⋅mol^−1^, Aladdin Industrial Corporation, Shanghai, China), absolute ethyl alcohol (≥99.7%, Sinopharm Chemical Reagent Co., Ltd, Shanghai, China), acetic acid (≥99.5%, Sinopharm Chemical Reagent Co., Ltd, Shanghai, China), sliver nitrate (AgNO_3_, ≥99.8%, Aladdin Industrial Corporation, Shanghai, China), diisopropyl azodiformate (DIPA, M_W_ ≈ 202.21 g⋅mol^−1^, Aladdin Industrial Corporation, Shanghai, China) were used to produce mesoporous TiO_2_ and Ag@TiO_2_ nanofibers. For the preparation of pristine TiO_2_ nanofibers, 0.35 g PVP was dissolved in 10 ml ethanol under continuous stirring for an hour. A total of 1.5 g acetic acid and 1.5 g TBOT were subsequently added into the above solution, finally 0.35 g DIPA was added into the solution. After stirring for 6 h, the slight yellow homogeneous solution was formed. For silver nanoparticles embedded into mesoporous TiO_2_ nanofibers, 0.05 g AgNO_3_ and 0.35 g DIPA were dissolved into the previous solution under stirring to form a bright yellow solution without precipitation. Before electrospinning, the precursor solution was moved into a 20 ml syringe with a stainless-steel needle (18 G). The aluminum foil was used to collect the nanofibers. The applied voltage between aluminum foil and needle point was controlled at 9 kV, and the distance was maintained about 9 cm. The electrospinning process can be intuitively described by a diagrammatic sketch (Figure 1). The obtained electrospun mats were detached from the collector and then annealed at 500 °C for 3 h in air. Finally, a white film (TiO_2_ nanofibers) and a grey film (mesoporous Ag@TiO_2_ nanofibers) were obtained for the experiments. 

### 2.2. Assembly of LIBs

TiO_2_ nanofibers (mesoporous Ag@TiO_2_ nanofibers), carbon black, and polyvinylidene fluoride (PVDF) with a mass ratio of 80:10:10 were added into N-methyl-2-pyrrolidone (NMP) to form the slurries. The resulting slurries were coated on the copper foils of 16 mm in diameter and dried at 60 °C for 12 h in a vacuum oven to obtain the TiO_2_ and mesoporous Ag@TiO_2_ working electrodes. The mass of the active material loading was about 2 mg, which was determined by the weight difference between the uncoated and the coated cooper foils. Pure lithium metal foils were used as the counter electrode. A solution of 1 M lithium hexafluorophosphate (LiPF_6_) in ethylene carbonate (EC) and diethyl carbonate (DEC) (1:1, v/v) was selected as the electrolyte. 2032-type coin cells were assembled in an argon-filled glovebox (Super 1220/750/900, Shanghai Mikrouna Electromechanical Technology Co., Ltd, Shanghai, China).

### 2.3. Characterizations

The morphologies and structures of the annealed samples were observed by a field-emission scanning electron microscope (FESEM, S-4800, HITACHI, Tokyo, Japan) and a transmission electron microscope (TEM, JEM-2100F, JOEL, Tokyo, Japan) coupled with an Energy Dispersive Spectrometer (EDS, X-MAX 65T, OXFORD, England, UK). Brunauer Emmett Teller (BET) nitrogen adsorption–desorption (JW-BK200B, Beijing JWGB Sci&Tech Co., Ltd., Beijing, China) at 77 K was used to measure specific surface areas of the samples. Their phase constituents were identified utilizing an X-ray diffractometer (XRD, D2-PHASER, Bruker, Karlsruhe, Germany) with Cu Kα radiation (γ = 0.1540560 nm). An X-ray photoelectron spectroscope (XPS, ESCALAB 250XI, Thermo Fisher Scientific, Waltham, MA, USA) was used to determine the main elements and their chemical valence states of the annealed samples.

Cyclic voltammetry (CV) tests were tested to understand the electrochemical behaviors by an electrochemical workstation (CHI 760E, CH Instruments Ins, Shanghai, China) at 0–3.0 V (vs. Li^+^/Li) at 0.1 mV/s. Glavanostatic charge-discharge tests were performed on an electrochemical workstation (CT4008, Neware Electronics Co., Ltd, Shenzhen, China). The cycle stability was assessed in the voltage range of 0.05 to 3 V (vs Li^+^/Li) at a current density of 100 mA·g^−1^ for 100 cycles. The rate capability was also evaluated at different current densities of 40, 100, 200, 400, and 1000 mA·g^−1^, respectively. Electrochemical impedance spectroscopy (EIS) was carried out to probe the kinetic properties of materials by an electrochemical workstation (Autolab-PGSTAT302N, Metrohm, Herisau, The Netherlands), which tested at room temperature with an AC amplitude of 10 mV applied over a frequency window of 0.1 MHz to 0.01 Hz.

## 3. Results and Discussion

### 3.1. Microstructural Characterization of Nanofibers

**Figure 2:**
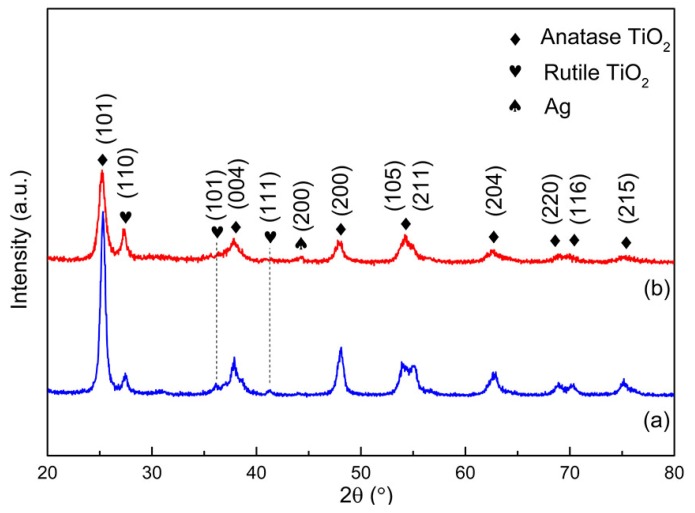
XRD patterns of (**a**) TiO_2_ nanofibers; (**b**) Ag nanoparticles-embedded TiO_2_ (Ag@TiO_2_) nanofibers.

Figure 2 indicates the XRD patterns of pure TiO_2_ and Ag loaded TiO_2_ nanofibers in the range of 20–80° (2θ). Some sharp peaks can be clearly observed, which implies that nanofibers have a good crystallinity after the annealing. Prior to the modification, the different peaks observed at 25.3°, 37.8°, 48.0°, 53.9°, 62.7°, 68.8°, 70.3°, and 75.0° are corresponded to the characteristic peaks of anatase TiO_2_ (JCPDS, no. 03-065-5714). The other weak peaks located at 27.4°, 35.9°, and 41.1° are related to rutile TiO_2_ (JCPDS, no. 03-065-1940). It can be concluded that the nanofibers are mainly composed of anatase TiO_2_, accompanied with traces of rutile TiO_2_. Anatase TiO_2_ can be formed by the reactions between the precursor containing Ti and oxygen at about 400 °C [13]. However, the product belongs to a kind of metastable phase in thermodynamics, which will be irreversibly transformed to rutile TiO_2_. This process is closely connected with temperature and time. A large temperature range from 400–1200 °C was reported to induce the transformation owing to the difference in raw material, preparation method, applied processing parameters, etc. The transformation also belongs to a time-dependent structural reconstruction process in which the breaking and reforming of bonds are involved [35]. Due to the comparatively low annealing temperature and short processing time, a large amount of anatase TiO_2_ was reversed.

When Ag is introduced into TiO_2_ nanofibers, the XRD pattern is very similar to that obtained in pure TiO_2_ nanofibers. However, clear inspection reveals that there are some tiny changes in number, width, and intensity of peaks. Two peaks at 35.9° and 41.4° related to rutile TiO_2_ completely disappear and a new peak identified as Ag appears at 43.8° (JCPDS, no. 03-065-8428). The peak associated with Ag from the decomposition of AgNO_3_ during annealing is very weak in intensity, which results from the slight content of Ag in nanofibers of about 11 wt %. AgNO_3_ will suffer from the decomposition at about 444 °C at atmospheric pressure. Some reports have proved that the addition of PVP in this study can promote the decomposition by significantly reducing the temperature to about 300 °C [36]. A close comparison indicates that the peaks related to TiO_2_ almost have no deviation in diffraction angle after the introduction of Ag, which further confirms that Ag mainly exists in the form of metallic silver without the solid solution in TiO_2_ lattice due to the significant difference in atomic radius between them (126 pm for Ag^+^ cations and 68 pm for Ti^4+^ cations). It is also worth noting that there are obvious increases in intensity ratio between two strong characteristic peaks in terms of the rutile (110) peak at 27.4° and the anatase (101) peak at 25.3°. The intensity ratio of the two peaks can be applied to empirically determine the weight fractions of anatase and rutile by the following formula [37]:

(1) $$X_{A}=\frac{100}{\left(1+1.265\frac{I_{R}}{I_{A}}\right)}$$

in which X_A_ is the percentage content of anatase phase, I_A_ and I_R_ are the integral intensities of anatase (101) and rutile (110) peaks, respectively. 

The calculated result indicates that the content of anatase is reduced from 87.1% to 71.2% after introducing Ag, implying that the addition of Ag can promote the transformation of TiO_2_ from anatase to rutile. Moreover, the anatase (101) characteristic peak at 25.2° becomes wider after introducing Ag, indicating that the addition of Ag contributed to refining anatase TiO_2_ grains. The above-mentioned two phenomena can be associated with the strengthening in heterogeneous nucleation resulting from the introduction of Ag. Ag is formed prior to anatase TiO_2_ during annealing owing to its lower reaction temperature of about 300 °C than that (about 400 °C) of the latter as mentioned above. Therefore, a large number of Ag nanoparticles distributed in the precursor will act as the heterogeneous nucleation sites and greatly facilitate the formation of anatase TiO_2_ nuclei, resulting in the refining of grains. The anatase to rutile transition will also undergo the nucleation and growth with prolonging the annealing time, during which Ag nanoparticles as the heterogeneous nucleation sites also play the positive role in phase transition. Consequently, the content of rutile is increased, accompanied with the grain refinement of anatase TiO_2_. 

In terms of TiO_2_ applied to the electrode material for lithium ion batteries, some investigations have confirmed that anatase TiO_2_ can provide higher lithium intercalation capacity at room temperature when compared with rutile TiO_2_ due to their special crystal structure with two-way channels along the a and b axes [38,39]. Therefore, promoting the transformation is not beneficial to the improvement in electrochemical performance of TiO_2_ electrodes. However, the subsequent experiments confirmed that the positive role in electrochemical performance resulting from Ag is sufficient to compensate for the negative role from the increase in content of rutile TiO_2_.

**Figure 3:**
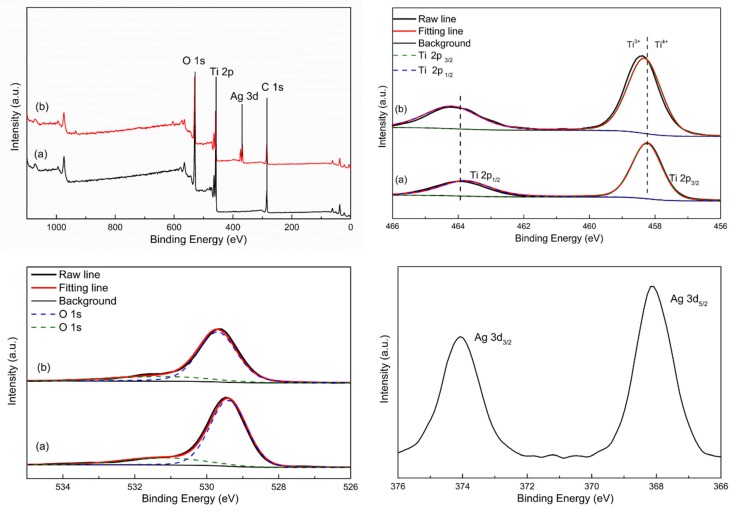
XPS survey spectra of (**a**) TiO_2_ nanofibers and (**b**) Ag@TiO_2_ nanofibers, and high-resolution XPS spectra of Ti 2p, O 1s, Ag 3d.

From the XRD pattern of mesoporous Ag@TiO_2_ nanofibers, the characteristic peaks of cubic Ag are not obvious. The elemental compositions and chemical valence states of Ti and Ag element involved in resulting samples were further ascertained by an X-ray photoelectron spectroscope (XPS). The representative XPS survey reveals that Ti, O, C elements exist in the TiO_2_ nanofibers (shown in Figure 3), while besides those peaks, a new peak related to Ag is clearly observed in the Ag@TiO_2_ samples. The presence of C 1s is ascribed to the samples contaminated with carbon from the XPS instruments. Two peaks corresponding to Ti 2p_3/2_ and Ti 2p_1/2_ are situated at 458.20 and 464.19 eV in the Ti 2p high-resolution spectrum of the pristine TiO_2_, which suggests the existence of Ti in TiO_2_. However, a strange phenomenon is noticed, namely that those peaks slightly move toward higher binding energy when Ag is introduced (Ti 2p_3/2_ and Ti 2p_1/2_ at 458.30 and 464.19 eV, O 1s at 531.2 and 529.4 eV). These slight changes may be caused by a tiny amount of Ti^3+^ oxide [40]. As far as the Ag 3d spectrum is concerned, two individual peaks with the binding energies of about 368.1 eV and 374.27 eV can be clearly ascertained, which can be assigned to Ag 3d_5/2_ and Ag 3d_3/2_ in metallic silver. The result is well in accordance with the XRD results.

**Figure 4:**
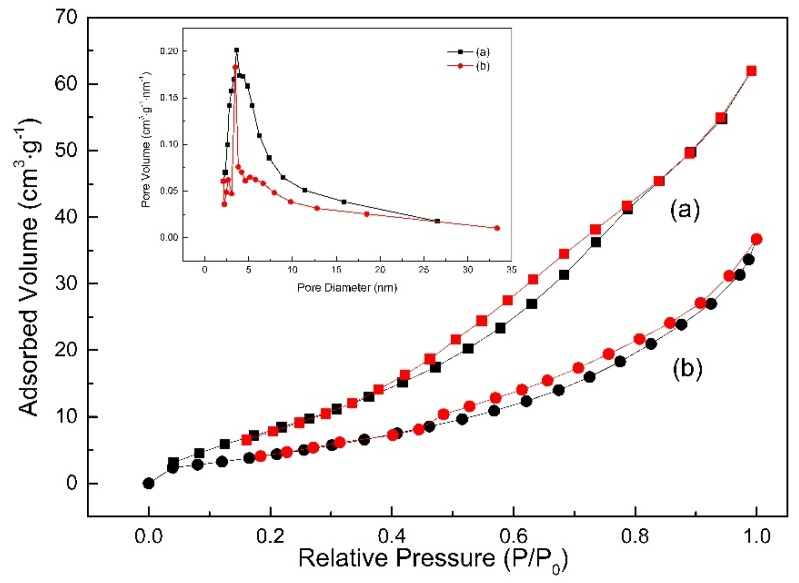
N_2_ adsorption–desorption isotherms of (**a**) TiO_2_ nanofibers and (**b**) Ag@TiO_2_ nanofibers.

Figure 4 shows the nitrogen adsorption–desorption isotherms of TiO_2_ and Ag@TiO_2_ nanofibers. Both samples exhibit the type IV pattern with a hysteresis loop in term of the Brunauer, Deming, Deming, Teller^3^ (BDDT) classification, demonstrating the typical characteristics of mesoporous materials. The Barrett Joyner Halenda (BJH) method was used to calculate pore size distributions from the adsorption branch of the Nitrogen adsorption–desorption isotherm. As shown in the inset in Figure 4, Ag@TiO_2_ nanofibers possess a slightly lower average pore diameter of 6.18 nm than that of TiO_2_ nanofibers (7.09 nm). The pore size of two nanofibers is mainly located at the range from 2 nm to 50 nm, between which the volume fraction reaches 92.58% (TiO_2_) and 87.27% (Ag@TiO_2_), respectively. Moreover, the introduction of Ag greatly reduces Brunner-Emmet-Teller (BET) surface area of TiO_2_ nanofibers from 41.2 m^2^⋅g^−1^ to 19.4 m^2^⋅g^−1^, which should be attributed to partial pores filled with Ag nanoparticles in Ag@TiO_2_ nanofibers and higher density of Ag than TiO_2_ (10.5 g⋅cm^−3^ for Ag and 4.0 g⋅cm^−3^ for TiO_2_).

**Figure 5:**
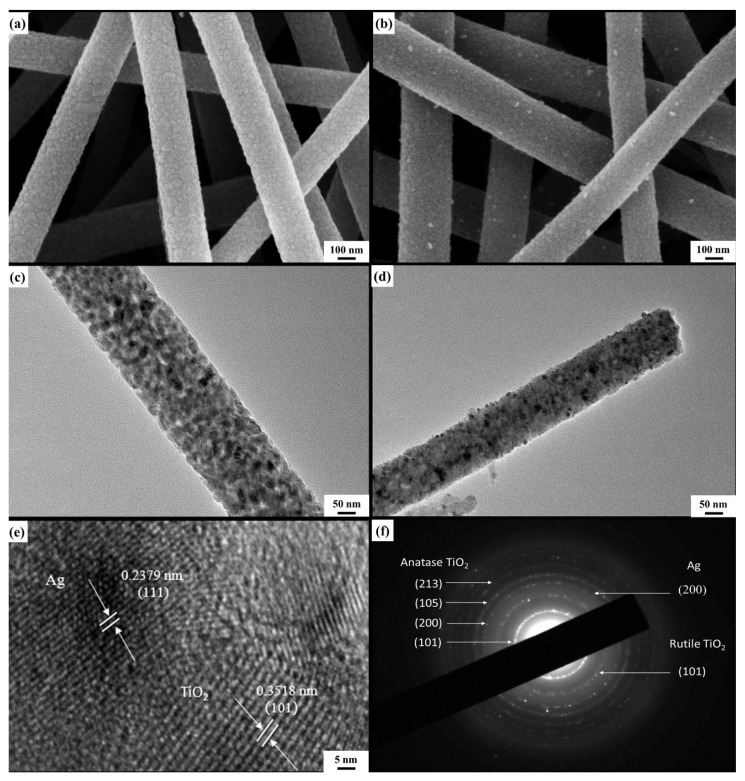
FE-SEM images of (**a**) TiO_2_ nanofibers, (**b**) Ag@TiO_2_ nanofibers. TEM images of (**c**) TiO_2_ nanofibers, (**d**) Ag@TiO_2_ nanofibers. (**e**) HRTEM image of a section of Ag@TiO_2_ nanofiber. (**f**) Selected-area electronic diffraction (SAED) pattern of Ag@TiO_2_ nanofibers.

**Figure 6:**
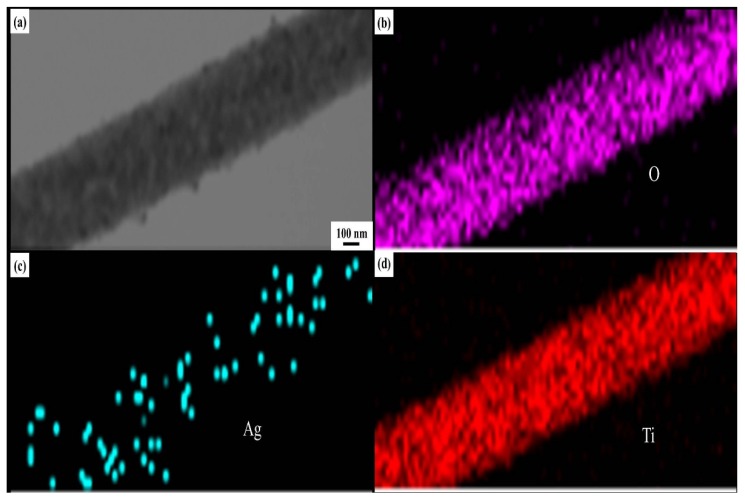
TEM image of (**a**) a single Ag@TiO_2_ nanofiber for elemental mapping. Mapping of (**b**) O, (**c**) Ag, and (**d**) Ti.

Detailed microstructure of the nanofibers with and without the introduction of Ag was examined by FE-SEM and TEM. As shown in Figure 5a,b, all of the nanofibers represent the continuous 1D structure with an average diameter of 100 ± 20 nm. Compared with the pure TiO_2_ nanofibers, some white nanoparticles scatter on the surfaces of Ag@TiO_2_ nanofibers, which indicate that Ag nanoparticles are successfully loaded in TiO_2_ nanofibers. TEM images further reveal that the surfaces of mesoporous Ag@TiO_2_ nanofibers are relatively smooth when compared with those of pure TiO_2_ nanofibers due to some mesoporous filled with fine Ag particles (Figure 5c,d). The lattice fringes are indicated in a HRTEM image (Figure 5e) in which the interplanar spaces of 0.2379 and 0.3518 nm could be identified to match with those of Ag (111) plane and anatase TiO_2_ (101) plane, respectively. This further provides a strong evidence for the existence of metallic Ag nanoparticles. The corresponding selected-area electronic diffraction (SAED) pattern (Figure 5f) taken from a single fiber displays six concentric diffraction rings. In terms of JCPDS cards, planes related to anatase TiO_2_, rutile TiO_2_, and sliver can be identified, which confirm the polycrystallinity form of three hybrid materials. The images of TEM examination combined with the corresponding EDS mapping for the O, Ag, Ti elements are showed in Figure 6a–d. Ti and O are almost distributed throughout the whole nanofibers; however, Ag nanoparticles with a diameter of about 6–10 nm are uniformly dispersed along the nanofiber direction, which would certainly provide a higher electronic conductivity.

### 3.2. Electrochemical Performance of the Nanofibers Electrodes

**Figure 7:**
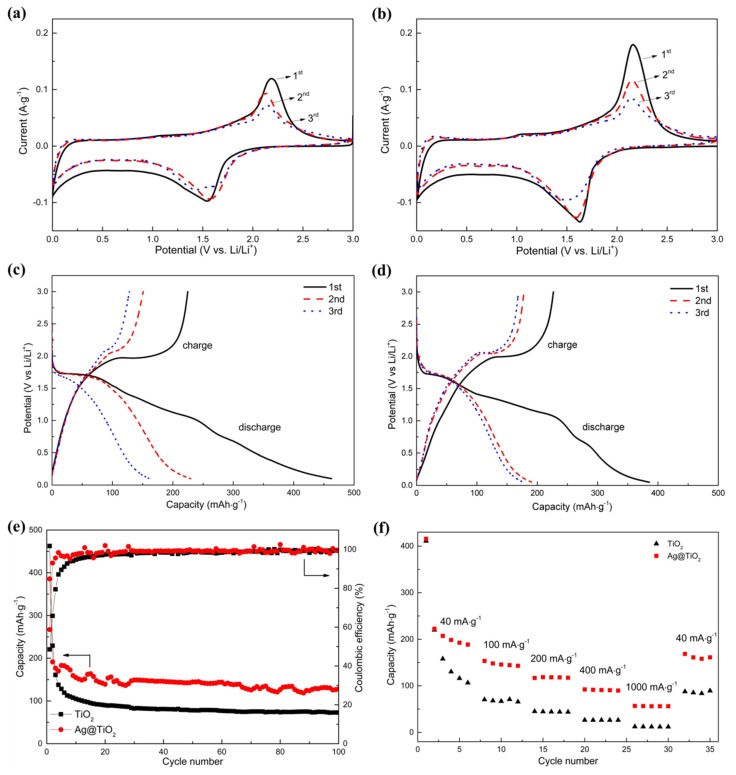
Electrochemical performances of TiO_2_ and Ag@TiO_2_ nanofibers electrodes. Cyclic voltammograms of (**a**) TiO_2_ nanofibers electrode and (**b**) Ag@TiO_2_ nanofibers electrode from the first to third cycle at a scanning rate of 0.1 mV/s between 0–3 V. First three charge and discharge cycles of (**c**) TiO_2_ nanofibers electrode and (**d**) Ag@TiO_2_ nanofibers electrode. (**e**) Cycling performance at 100 mA⋅g^−1^. (**f**) Rate capability of TiO_2_ and Ag@TiO_2_ nanofibers electrodes at different current densities.

Figure 7a,b compares the representative cyclic voltammograms (CV) of the TiO_2_ and Ag@TiO_2_ nanofibers electrodes from the first to the third cycle at a scanning rate of 0.1 mV/s in the voltage range of 0–3 V (vs Li^+^/Li). A pair of similar redox peaks resulting from Li insertion into anatase TiO_2_ and Li^+^ desertion from anatase TiO_2_ can be observed at about 1.68 and 2.00 V (vs Li^+^/Li), which can be described by the following intercalation-type action:

(2) $${\mathrm{TiO}}_{2}+{\mathrm{xLi}}^{+}+{\mathrm{xe}}^{-}↔{\mathrm{Li}}_{x}{\mathrm{TiO}}_{2}$$

in which x denotes the lithium ion insertion coefficient (x is close to 0.5 for the anatase structure when it is at max accommodation [41,42]). 

No extra peaks appear when Ag is introduced into TiO_2_ nanofibers, suggesting that Ag serves as an inert material and is not involved in the electrode reactions during charging and discharging. Moreover, the addition also causes the sharp increase in current density of the TiO_2_ nanofibers electrode at the same applied potential, which confirms that Ag contributes to the improvement in transfer rate of charges. The diffusion coefficient of samples can be calculated by the Randles-Sevcik equation (shown as following) [22,43]:

(3) $$I_{p}=0.4463\times {\left(F^{3}/\mathrm{RT}\right)}^{1/2}\times n^{3/2}\times S\times C_{\mathrm{Li}}\times v^{1/2}\times {D_{\mathrm{Li}}}^{1/2}$$

in which I_p_ is the peak current (A), F is the Faraday constant (C⋅mol^−1^), R is the gas constant (J mol^−1^⋅K^−1^), T is the temperature (K), n is the number of electrons transferred, S is the contact area between the electrode and electrolyte (cm^2^), С_Li_ is the concentration of Li ion (mol⋅cm^−3^), ν is the scan rate (V⋅s^−1^), D_Li_ is the diffusion coefficient (cm^2^⋅s^−1^).

The calculated results indicate that the diffusion coefficient of Ag@TiO_2_ (1.96 × 10^−8^ cm^2^⋅s^−1^) is significantly higher than that of pristine TiO_2_ (5.42 × 10^−9^ cm^2^⋅s^−1^) at the first cycle.

The galvanostatic charge and discharge curves for chief three cycles of TiO_2_ and Ag@TiO_2_ nanofibers at a current density of 100 mA⋅g^−1^ in a voltage of 0–3.0 V are revealed in Figure 7c,d. The electrode consisting of pristine TiO_2_ nanofibers (Figure 7c) exhibited first discharge and charge capacities of 462.67 and 224.67 mAh⋅g^−1^, respectively, with the initial coulombic efficiency of 48.56%. There are two distinct voltage plateaus at about 1.75 and 1.98 V during the discharge (Li insertion) and charge (Li extraction) process, which agree well with the above-mentioned redox peaks in CV curves. With the increase in cycling number, the discharge and charge capacities are decayed to 229.35 and 151.00 mAh⋅g^−1^ (the second cycle), finally to 161.02 and 128.02 mAh⋅g^−1^ (the third cycle). However, the coulombic efficiency is improved to 65.8% and 79.5%, respectively. The initial capacity loss can be ascribed to the formed solid electrolyte interface (SEI) layer on the surface of active material resulting from the decomposition of the electrolyte during the first discharge process [44]. The formation of this layer will consume the considerable Li ions during Li insertion (the specific capacity is even higher than the theory value of about 335 mAh⋅g^−1^) [17], however those Li ions will be pinned in the SEI layer lattice during Li extraction, resulting in the serious loss in specific capacity in the first cycle [20]. The SEI layer tends to be stable during subsequent discharging and charging so that less and less Li ions are embedded, causing the gradual increase in coulombic efficiency. For the Ag@TiO_2_ nanofiber electrodes, the first three charge and discharge curves are similar to the pristine TiO_2_ without the other charge/discharge plateaus, suggesting that the sliver nanoparticles just play an essential role in improving the electrical conductivity of pristine TiO_2_ electrode. Moreover, it is worth noting that the second and third cycle curves almost coincide with each other, which can be clearly distinguished in the TiO_2_ nanofibers electrode. Its first discharge and charge capacities are 385.75 and 226.77 mAh⋅g^−1^, and the values are gradually decreased to 191.47/178.06 mAh·g^−1^ and 176.83/169.01 mAh·g^−1^ for the second and third discharge and charge, respectively. The initial coulombic efficiency of the Ag@TiO_2_ nanofiber electrode is about 58.79% and rapidly enhanced to 92.96% and 95.58% for the second and third discharge and charge.

**Figure 8:**
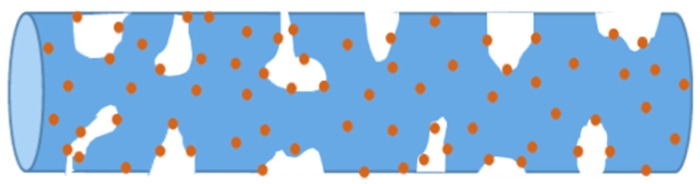
Schematic illustration of the distribution of Ag nanoparticles in Ag@TiO_2_ nanofibers.

By comparing the above-mentioned data, the following changes from the addition of Ag can be revealed: (1) The discharge capacities of the TiO_2_ nanofibers electrode are higher than those with the addition of Ag at the first two discharges, and then the opposite change is observed when the electrodes suffer from the third charge and discharge. (2) The charge capacities of the TiO_2_ nanofibers electrode are all far less than those with the addition of Ag. The charge and discharge capacities are closely associated with the two existence forms of Ag. As shown in Figure 8, a portion of Ag particles may be surrounded by TiO_2_ and cannot directly contact with the electrolyte. The other particles may adhere to the TiO_2_ surfaces, resulting in their partial surfaces directly exposed to the electrolyte. For the former, the occupation of Ag without chemical activeness has no the function of accommodating Li ions, which causes the reduction in discharge specific capacity (Effect 1). However, the addition of Ag with a high conductivity also accelerates the diffusion of Li ions in the TiO_2_ nanofibers, which is beneficial to the improvement in discharge specific capacity (Effect 2). For the latter, Ag particles as the barriers shield partial TiO_2_ surfaces from the contact with the electrolyte and delay the diffusion of Li ions into the electrode (Effect 3). Moreover, some particles may be regarded as the channel-blocking point defects, which postpone the diffusion of electrolyte into the internal zones of the pores (Effect 4). During the initial discharge, Effects 1, 3, and 4 are predominant, so that the specific discharge capacity of the electrode with the addition of Ag is obviously reduced when compared with that without Ag. However, Effect 4 is slowly weakened and finally removed due to the internal zones of the pores gradually activated along with the cycling discharging, accompanied with which Effect 2 intends to play a leading role in the improvement in specific discharge capacity. Consequently, the difference in specific discharge capacity of two electrodes is shortened in the second discharge and the specific discharge capacity of the electrode with Ag surpasses that without Ag at the third circle. During charging, the increase in specific charge capacity of the electrode with Ag is mainly attributed to the significant improvement in transfer rate of electrons and Li ions due to high conductivity of Ag. The change in specific discharge/charge capacity of the two electrodes results in the coulombic efficiency of the electrode with Ag higher than that without Ag and rapidly close to 100% in a shorter time. 

The cycling stability of the two electrodes subject to 100 cycles was also investigated. As shown in Figure 7e, the coulombic efficiency of the electrode with Ag is improved to 95.58% in the third cycle, and finally retains about 99.80% with a comparatively high specific discharge capacity of about 127.97 mAh⋅g^−1^ after 100 loops. However, the coulombic efficiency of the electrode without Ag reaches 95.25% after undergoing 12 cycles, and finally retains about 99.39% with specific discharge capacity of about 72.32 mAh⋅g^−1^. The delay in specific discharge capacity of the TiO_2_ electrode along cycling may be related to the inactivation of a portion of inactive Li^+^ ions embedded into the inside lattices, which is difficult to be efficiently released from TiO_2_ with a low conductivity (10^−7^–10^−9^ S⋅cm^−1^) [15] during cycling. The ability of Li^+^ desertion and insertion are improved with the addition of high-conductive Ag, resulting in the improvement in reversible capacity and cycling stability. 

Rate performances of these two samples were explored at different current density (Figure 7f). With decreasing the current density in turn (40, 100, 200, 400, 1000 mA·g^−1^), the average specific discharge capacity of the TiO_2_ nanofibers electrode was drastically reduced from 156.02 mAh·g^−1^ to 12.14 mAh⋅g^−1^, with a capacity retention of only 7.78%. However, about 27.91% of the initial value of 201.89 mAh⋅g^−1^ is retained (56.35 mAh·g^−1^) along the introduction of Ag, which is approximately five times that without Ag. When the current density is returned to the initial value (40 mA·g^−1^), the specific discharge capacity of the electrode with Ag is returned to 80.36% of the initial value (162.25 mAh·g^−1^), which is about twice of the value obtained in the electrode without Ag (86.50 mAh·g^−1^). Clearly, the mesoporous Ag@TiO_2_ nanofibers electrode exhibits better rate capability. 

The electrochemical performance of Ag@TiO_2_ nanofibers obtained in our research is also compared with that of TiO_2_ composites reported in References [23,31,32,33,34,43,45,46]. As shown in Table 1, the comprehensive electrochemical performance obtained in our research is generally superior to that reported in the above-mentioned references. The discharge/charge capacity is 385/226 mAh·g^−1^ at 100 mA·g^−1^, which is higher than that reported in the above references. The Ag@TiO_2_ nanofibers electrode subject to 100 cycles at 100 mA⋅g^−1^ still retains a high discharge capacity of 128 mAh⋅g^−1^, which exhibits a better cycling stability than the reported electrodes in the above references. The Ag@TiO_2_ nanofibers electrode suffering from discharging/charging at different current densities (40 mA⋅g^−1^, 100 mA⋅g^−1^, 200 mA⋅g^−1^, 400 mA⋅g^−1^, 1000 mA⋅g^−1^) still demonstrates a more excellent rate capability due to a higher discharge capacity of 56 mAh⋅g^−1^ remained when compared with the reported electrodes. Although the electrochemical performance of Ag@TiO_2_ nanofibers electrode prepared in this study does not reach the commercial level, the improved electrochemical performance will promote TiO_2_ electrodes closer to the commercial application. Moreover, the new strategy proposed in this study will provide a new choice for synthesizing the TiO_2_ electrode with excellent electrochemical performance. 

**Figure 9:**
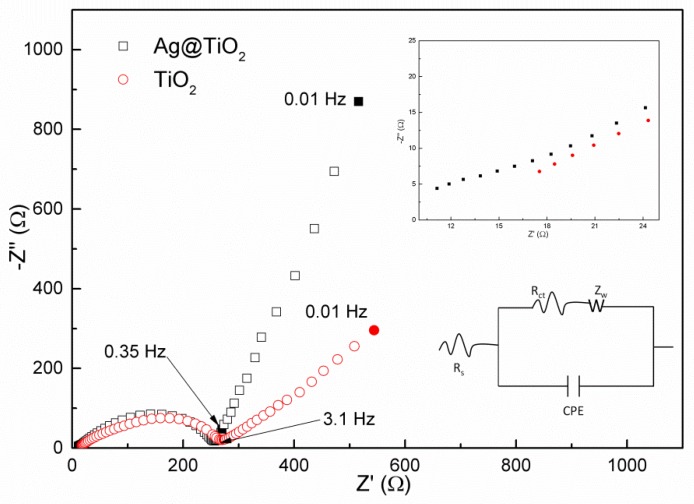
Nyquist plots of pristine TiO_2_ nanofibers electrode and Ag@TiO_2_ nanofibers electrode at room temperature.

The above results imply that the incorporation of Ag particles into the TiO_2_ remarkably improves the comprehensive electrochemical performance in terms of the specific capacity, coulombic efficiency, cycling stability, and rate capability due to their promotion in transfer rate of electrons and Li ions. The conjecture can be further confirmed by electrochemical impedance spectroscopy (EIS). It is well known that EIS is considered as a powerful method to probe the kinetic properties of materials. Figure 9 shows the obtained EIS data and fitting results using an equivalent circuit of the two electrodes, which were measured at the open circuit potential of 2.8 V. The spectra are composed of a semicircle at a high frequency followed by an inclined tail at a low frequency. The intercept of the semicircle with the real axis is associated with the ohmic resistance (R_s_) in which the resistance of electrolyte, SEI film, and electrode is involved. The radius of the high frequency semicircle reflects the charge-transfer resistance (R_ct_) and the slope of the straight line corresponds to Warburg impedance of the lithium ion diffusion toward the electrode [47,48]. The drawing of partial enlargement clearly illustrates that the R_s_ of the TiO_2_ electrode is 11.9 Ω smaller than of that without the addition of Ag (17.6 Ω). The introduction of Ag also causes the slight decrease in radius of the high frequency semicircle, showing that R_ct_ is reduced from 265 Ω to 225 Ω for the first cycle. Additionally, the obvious increase in slope of the straight line clearly demonstrates that the resistance of Li ion diffusion into the electrode is obviously reduced resulting from the introduction of Ag [49]. According to EIS data, the conductivity of TiO_2_ and Ag@TiO_2_ can be calculated [50]. The results indicate that it is 1.69 × 10^−5^ S·cm^−1^ and 1.99 × 10^−5^ S·cm^−1^, respectively. It is clear that the introduction of Ag nanoparticles with a high conductivity is beneficial to the improvement in conductivity of TiO_2_. The results agree well with those obtained in electrochemical measurement.

## 4. Conclusions

In summary, mesoporous Ag@TiO_2_ nanofibers were successfully synthesized using an electrospinning method followed by annealing method. As mentioned above, sliver nanoparticles incorporated uniformly in the TiO_2_ nanofibers. These mesoporous Ag@TiO_2_ nanofibers electrodes displayed a high initial discharge capacity of 385.75 mAh·g^−1^ and delivered a high reversible capacity of approximately 127.97 mAh·g^−1^ after 100 cycles at 100 mA·g^−1^ with a high coulombic efficiency of nearly 100%. The electrode also demonstrated an excellent rate capability. With the current density increased from 40 mA⋅g^−1^, 100 mA⋅g^−1^, 200 mA⋅g^−1^, 400 mA·g^−1^, finally to 1000 mA·g^−1^ in turn, the higher average discharge capacity of 56.35 mAh⋅g^−1^ was remained in the electrode with Ag, when compared with the electrode without Ag (average discharge capacity of about 12.14 mAh⋅g^−1^). When the current density was returned to 40 mA⋅g^−1^, 80.36% of the initial value was returned (about 162.25 mAh·g^−1^) in the electrode with Ag, which was evidently superior to that without Ag (about 86.50 mAh·g^−1^, only 55.42% of the initial value). The above improvement in electrochemical performance should be attributed to the enhancement in the diffusion coefficient of Li ions (5.42 × 10^−9^ cm^2^·s^−1^ for pristine TiO_2_, 1.96 × 10^−8^ cm^2^·s^−1^ for Ag@TiO_2_) and the electronic conductivity of TiO_2_ (1.69 × 10^−5^ S·cm^−1^ for pristine TiO_2_ and 1.99 × 10^−5^ S⋅cm^−1^ for Ag@TiO_2_). All these results indicate that mesoporous Ag@TiO_2_ nanofibers can be chosen as a candidate for a potential anode material for lithium ion batteries. 

## Figures and Tables

**Table 1:** The discharge/charge capacity in 1st cycle, cycle performance and rate performance of multicomponent nanostructured TiO_2_ composites reported in some related references.

Ref.	Materials	Discharge/Charge Capacity	Cycle Performance	Rate Performance
Our research	Ag@TiO_2_ nanofibers	385/226 mAh·g^−1^ at 100 mA·g^−1^	128 mAh·g^−1^/100 cycles at 100 mA·g^−1^	56 mAh·g^−1^/1000 mA·g^−1^
[23]	Nb@TiO_2_ nanofibers	252/115 mAh·g^−1^ at 16.8 mA·g^−1^	–	20 mAh·g^−1^/1500 mA·g^−1^
[31]	Al@TiO_2_ nanofibers	225/175 mAh·g^−1^ at 66 mA·g^−1^	150 mAh·g^−1^/100 cycles at 40 mA·g^−1^	50 mAh·g^−1^/1000 mA·g^−1^
[32]	Au@TiO_2_ nanofibers	180/120 mAh·g^−1^ at 66 mA·g^−1^	150 mAh·g^−1^/50 cycles at 66 mA·g^−1^	48 mAh·g^−1^/1600 mA·g^−1^
[34]	3D-Ag@TiO_2_ nanotubes	180/90 mAh·g^−1^ at 0.3 C	90 mAh·g^−1^/50 cycles at 0.3 C	90 mAh·g^−1^/2 C
[43]	Hf@TiO_2_ nanofibers	320/160 mAh·g^−1^ at 33.5 mA·g^−1^	170 mAh·g^−1^/35 cycles at 33.5 mA·g^−1^	50 mAh·g^−1^/335 mA·g^−1^
[45]	TiO_2_/graphene nanosheets	135/82 mAh·g^−1^ at 50 mA·g^−1^	120 mAh·g^−1^/40 cycles at 200 mA·g^−1^	100 mAh·g^−1^/500 mA·g^−1^
[46]	Cu/Ni/TiO_2_ nanofibers	280/250 mAh·g^−1^ at 33.5 mA·g^−1^	110 mAh·g^−1^/30 cycles at 33.5 mA·g^−1^	20 mAh·g^−1^/670 mA·g^−1^
